# Negatively Skewed Locomotor Activity Is Related to Autistic Traits and Behavioral Problems in Typically Developing Children and Those With Autism Spectrum Disorders

**DOI:** 10.3389/fnhum.2018.00518

**Published:** 2018-12-21

**Authors:** Kazuo Ogino, Hidetoshi Takahashi, Toru Nakamura, Jinhyuk Kim, Hiroe Kikuchi, Takayuki Nakahachi, Ken Ebishima, Kazuhiro Yoshiuchi, Tetsuya Ando, Tomiki Sumiyoshi, Andrew Stickley, Yoshiharu Yamamoto, Yoko Kamio

**Affiliations:** ^1^National Center of Neurology and Psychiatry, Department of Preventive Intervention for Psychiatric Disorders, National Institute of Mental Health, Tokyo, Japan; ^2^Integrative Brain Imaging Center, Department of Advanced Neuroimaging, National Center of Neurology and Psychiatry, Tokyo, Japan; ^3^Graduate School of Education, The University of Tokyo, Tokyo, Japan; ^4^Department of Psychosomatic Medicine, Center Hospital of the National Center for Global Health and Medicine, Tokyo, Japan; ^5^Department of Stress Sciences and Psychosomatic Medicine, Graduate School of Medicine, University of Tokyo, Tokyo, Japan; ^6^National Center of Neurology and Psychiatry, Department of Psychosomatic Research, National Institute of Mental Health, Tokyo, Japan; ^7^Stockholm Center for Health and Social Change (SCOHOST), Södertörn University, Huddinge, Sweden

**Keywords:** autism spectrum disorders, autistic traits, social awareness, hyperactivity/inattention, locomotor activity, quantitative behavioral phenotypes

## Abstract

An important objective for researchers and clinicians is to gain a better understanding of the factors that underlie autism spectrum disorders (ASDs). It is possible that investigating objective and quantitative behavioral phenotypes and their relationship to clinical characteristics, such as autistic traits and other emotional/behavioral problems, might facilitate this process. Given this, in the current study we examined the link between locomotor dynamics and clinical characteristics, including autistic traits and emotional/behavioral problems, in children with ASD (*n* = 14) and typically developing (TD) children (*n* = 13). A watch-type actigraph was used to continuously measure locomotor activity which was assessed in terms of mean activity levels and the skewness of activity. Parents assessed quantitative autistic traits using the Japanese version of the Social Responsiveness Scale (SRS) and emotional and behavioral problems using the Japanese version of the Strengths and Difficulties Questionnaire (SDQ). Results showed that among all children, all-day activity was more negatively skewed, suggesting sporadic large all-day “troughs” in activity and was significantly correlated with the SRS social awareness subscale score (*ρ* = −0.446, *p* = 0.038). In addition, the more negatively skewed daytime locomotor activity was associated with the SDQ Hyperactivity Inattention subscale score (*ρ* = −0.493, *p* = 0.020). The results of this study indicate that investigating locomotor dynamics may provide one way to increase understanding of the neurophysiological mechanisms underlying the clinical characteristics of ASD.

## Introduction

The potential importance of translational research for autism spectrum disorder (ASD) is increasing given that its biological pathology and fully effective treatments have not yet been determined. Specifically, the acquisition of more knowledge about objective and quantitative neurobiological and behavioral indices may facilitate the development of basic and clinical research as well as lead to possible ASD phenotypes being identified.

In this context, locomotor dynamics may constitute a useful objective and quantifiable measure for translational research, as investigation of this behavioral index is less invasive and can be continued for an extended period of time in both laboratory (animal) and clinical research for the study of psychiatric and developmental disorders (Nakamura et al., 2008). Focusing on higher-order statistics such as skewness might be especially useful when characterizing behavioral alterations in psychiatric and developmental disorders. For example, we recently reported that in children with ASD, locomotor activity was significantly more negatively skewed—defined as a left-skewed distribution (or a long left tail relative to the right tail) with extreme values lower than their mean—for all-day activity, and also tended to be more negatively skewed for daytime activity, and, that this pattern of locomotor activity was related to acoustic hyper-reactivity (Takahashi et al., 2018). Further extending the use of actigraphy to help determine the clinical relevance of locomotor activity in ASD may help reveal important mechanisms in the underlying neurophysiology of this condition, while using higher-order statistics might further contribute to the realization of this purpose.

The objective of this study therefore was to investigate the relationship between locomotor activity and different clinical characteristics to determine the clinical relevance of locomotor activity in ASD and typically developing (TD) children. More specifically, as the investigation of locomotor activity is more common in attention-deficit hyperactivity disorder (ADHD; Cheung et al., 2015, 2016; De Crescenzo et al., 2016) compared to ASD, and ASD is known to have several comorbid psychiatric and developmental problems including attention deficit and hyperactivity (Lai et al., 2014), in this study we investigated the relationship between locomotor activity and emotional/behavioral problems as well as autistic traits. We hypothesize that locomotor activity indexes measured in daily life, such as negative skewness—which are related to ASD—will also be related to these clinical characteristics of ASD.

## Materials and Methods

### Participants

Data were used from 27 Japanese children aged from 7 to 16 years old. Fourteen had ASD (13 boys) while 13 were TD children (10 boys). All participants were included in our previous study (Takahashi et al., 2018). Diagnoses were made by child psychiatrists after medical records were reviewed and a clinical interview had been performed based on the Diagnostic and Statistical Manual of Mental Disorders, Fourth Edition, Text Revision (American Psychiatric Association, 2000). Diagnoses were confirmed by using the Autism Diagnostic Observation Schedule (Lord et al., 2000) and Autism Diagnostic Interview-Revised (Lord et al., 1995). There were no significant differences between the groups in terms of sex, age (age in months; ASD 125.6 ± 30.9; TD 138.5 ± 38.2; *U* = 76, *p* = 0.467). The Wechsler Intelligence Scale for Children-Third Revision (Wechsler, 1991) showed that, the estimated IQ was above 70 for every child included in the study, and did not differ between groups (IQ: ASD 105.7 ± 23.3; TD 104.7 ± 18.3; *U* = 31, *p* = 0.958). No children were being medicated with psychotropic substances and none of them smoked. Besides having autism, no children had any central nervous system abnormalities. For the TD group having a previous or current psychiatric diagnosis or learning disability served as exclusion criteria.

### Ethics Approval and Consent to Participate

This study was conducted in accordance with the Helsinki Declaration with institutional review-board approval being granted by the Research Ethics Committee of the National Center of Neurology and Psychiatry (#A2013-112) and the research ethics committee of the Graduate School of Education, the University of Tokyo (#13-119). After the details of the study were explained to them, all participants and their parents provided written informed consent before being included in the study.

### Assessment of Autistic Traits and Emotional/Behavioral Problems

Parents used the Japanese version (Kamio et al., 2013) of the Social Responsiveness Scale (SRS; Constantino and Gruber, 2005) to assess quantitative autistic traits. This scale consists of five treatment subscales: social awareness, social cognition, social communication, social motivation, and autistic mannerisms. Higher SRS scores indicate increased social impairment.

To assess emotional and behavioral problems, we used the Japanese version (Moriwaki and Kamio, 2014) of the Strengths and Difficulties Questionnaire (SDQ; Goodman, 1997). This measure also has five subscales: four difficulty subscales (Emotional Symptoms, Conduct Problems, Hyperactivity Inattention, and Peer Problems) and one strengths subscale (Prosocial Behavior). The scores of the four difficulty subscales can be summed to create a total difficulties score. As with the overall SDQ difficulties score, higher scores on the individual difficulty subscales reflect greater difficulties. In contrast, higher scores on the prosocial behavior subscale indicate increased prosociality.

### Assessment of Locomotor Dynamics

In order to assess locomotor dynamics participants were informed that they should wear the MicroMini Motionlogger actigraph (Ambulatory Monitors Inc., Ardsley, NY, USA; Teicher, 1995) on the wrist of their non-dominant hand. Specifically, that they should wear it on their wrist for > 7 days during the spring, summer, or winter school vacations (TD: 7.7 ± 1.9 days, ASD: 7.8 ± 1.8 days, *U* = 80, *p* = 0.574). Full details of the assessment of locomotor dynamics are provided in our previous study (Takahashi et al., 2018). It was expected that children would wear this device continuously throughout the study period. However, they were instructed to remove it when either bathing, or during rigorous exercise, or when performing any activity that might result in the device being damaged. They were also informed that they should just lead their lives normally while wearing the actigraph device. After the recording period had finished, three boys with ASD and two boys with TD had to be excluded from the actigraph behavioral data analysis for not wearing the actigraph for a long enough period of time during the daytime hours.

As described previously (Takahashi et al., 2018), sleep–wake cycles were scored using Action W-2 software, and we now further examine the mean and skewness, the first- and third-order statistical moments, of the locomotor distributions, for all-day and daytime activity. Sleep parameters were not examined, since none of them differed significantly between ASD and TD children.

### Statistical Analysis

To examine categorical differences in the participants’ scores we used Chi-square tests (and Fisher’s exact tests when necessary). Due to the non-normal distributions of most of the locomotor activity and clinical characteristic variables nonparametric analyses were performed. The Mann-Whitney U test was used to compare mean parameter values. Spearman’s rank order correlation coefficients were used to examine the relationships between variables. The level of statistical significance was set at *p* < 0.05. As multiple tests were performed a Bonferroni adjustment was subsequently applied to determine statistical significance. SPSS Ver. 22 (IBM Japan, Tokyo, Japan) was used to perform all statistical analyses.

## Results

### Differences in Clinical Characteristics Between Children With Autism Spectrum Disorders and Controls

All SRS scores were significantly higher in the ASD group than in the controls (Table 1). Compared with the TD group, the ASD group had a significantly lower SDQ Prosocial Behavior score and significantly higher scores for Total Difficulties and the SDQ Hyperactivity Inattention and Peer Problems subscales (Table 1).

**Table 1:** Clinical characteristics of the participants.

	Typical development		Autism spectrum disorders			
	*Mean*	*SD*	*Mean*	*SD*	*U*	*P*
Social Responsiveness Scale						
Total Score	20.5	10.0	70.1	34.4	8	<0.001
Social Awareness	3.2	2.3	9.9	3.5	2	<0.001
Social Cognition	4.0	3.2	14.6	5.4	7.5	<0.001
Social Communication	6.6	3.9	22.2	13.7	16.5	<0.001
Social Motivation	4.3	3.6	8.7	6.3	49	0.043
Autistic Mannerisms	2.5	1.5	14.7	9.1	12	<0.001
Strengths and Difficulties Questionnaire						
Total Difficulties Score	6.3	3.7	15.8	6.6	17.5	<0.001
Emotional Symptoms	1.2	1.7	2.9	2.3	52.5	0.610
Conduct Problems	1.6	1.4	2.9	2.0	57	0.105
Hyperactivity Inattention	2.3	0.9	5.9	2.3	19	<0.001
Peer Problems	1.2	1.2	4.1	2.8	32.5	0.003
Prosocial Behavior	7.7	1.4	4.9	2.9	33	0.004

*SD, standard deviation; Mann-Whitney U test. Number of participants (typical development: autism spectrum disorders) = 13:14*.

### Relationship of Locomotor Dynamics to Clinical Characteristics

**Figure 1:**
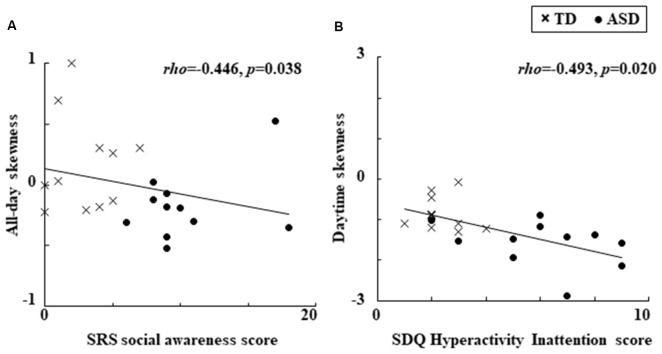
Significant relationships observed between locomotor dynamics and clinical characteristics. **(A)** Relationship between all-day skewness and the SRS social awareness score. **(B)** Relationship between daytime skewness and the SDQ Hyperactivity Inattention score. ASDs, Autism Spectrum Disorders; SDQ, Strengths and Difficulties Questionnaire; SRS, Social Responsiveness Scale; TD, Typical Development.

We found significant relationships between the locomotor dynamics and clinical characteristics which are presented in Figure 1. Specifically, for all children combined there was a significant negative relationship between all-day skewness and the SRS social awareness score (Figure 1A). In addition, there was also a significant negative relationship between daytime skewness and the SDQ Hyperactivity Inattention score (Figure 1B). No other significant relationships were observed for the clinical characteristics of locomotor dynamics, while the significant relationships described above became non-significant when we divided the children into groups (ASD and TD).

## Discussion

The results of this study showed that more negatively skewed all-day locomotor activity evaluated with an actigraph was related to the SRS social awareness score when the ASD and TD children were combined in one group. In addition, more negatively skewed locomotor activity during the daytime was related to the SDQ Hyperactivity Inattention score. To the best of our knowledge, this is the first time an association has been reported between locomotor dynamics and clinical characteristics, including autistic traits and emotional/behavioral problems, in children with ASD and TD. Our results suggest that besides using standard descriptive statistics when examining behavioral characteristics in ASD children, it may also be beneficial to utilize third-order statistical moments such as skewness.

For all children combined, the autistic trait of social awareness was related to significantly more negative skewness in all-day locomotor activity (defined as a left-skewed distribution with extreme values lower than their mean, which suggests behavior marked by an increase in large sporadic “troughs” below mean activity levels), with this behavioral characteristic having been previously found in ASD compared to TD children (Takahashi et al., 2018). Although the biological background of social awareness is uncertain, it might be associated with von Economo’s neurons, which are also known to be involved in motor awareness (Cauda et al., 2014). Thus, the use of higher order statistics, such as skewness to examine locomotor activity in animals and humans might help clarify the biological background of autistic traits, including social awareness.

The behavioral problem of hyperactivity/inattention was related to significantly more negative skewness of daytime locomotor activity, which tended to be more negative in children with ASD compared to control children (Takahashi et al., 2018). These results support the idea that ADHD may be highly comorbid in ASD (Lai et al., 2014), and suggest daytime locomotor activity and its skewness might serve as a potential behavioral phenotype that is connected with the comorbid clinical features of hyperactivity/inattention in ASD children. Several comorbid problems are frequently reported in ASD (Lai et al., 2014), including hyperactivity, inattention as well as motor abnormalities, such as motor delay, deficits in coordination and movement planning. Atypical movement in ASD may be regulated by emotion (Trevarthen and Delafield-Butt, 2013; Vernazza-Martin et al., 2013). Future studies investigating these comorbid features in relation to locomotor activity and emotional regulation in daily life might help elucidate the neurophysiological mechanisms that may underlie these features in ASD.

The small number of children in the ASD and TD groups is a significant study limitation. While it was still possible to detect significant associations between some aspects of locomotor dynamics and clinical characteristics for all children combined, significant relationships were not observed when the children were divided into groups. Further, gender differences exist in many aspects of ASD (Lai et al., 2014), however, participants in this study were mainly boys, while the age span was rather large. In addition, although we did not find significant differences in sleep parameters in our previous study (Takahashi et al., 2018), sleep problems are frequently observed in ASD children (Lai et al., 2014) and might possibly be seasonal (Hayashi, 2001), which could have impacted on our analysis of both nighttime and daytime locomotor activity. Given this, research that uses a larger number of children of both sexes with a narrower age range that also controls for season is now warranted to more clearly determine the relationship between locomotor dynamics and different clinical characteristics.

## Conclusion

Negatively skewed all-day locomotor activity (as seen in activity that was characterized by large sporadic all-day “troughs,”) might be a potentially useful quantitative behavioral index related to ASD, especially autistic social awareness. For all children, more negatively skewed daytime locomotor activity was also associated with comorbid hyperactivity/inattention behavioral problems. The results of this study thus build on and extend previous research on locomotor dynamics and further develop understanding of the potential neurophysiological mechanisms that may underlie clinical characteristics in ASD.

## Data Availability

The datasets generated for this study are available on request to the corresponding author.

## Author Contributions

HT, ToN, JK, HK, KY, TA, YY, and YK conceived and designed the experiments. HT, ToN, YY, and YK supervised the project. KO, HT and YK confirmed diagnoses. KO, HT, ToN, JK, and TaN performed the experiments. KO, HT, ToN, JK, YY, and YK analyzed the data. KO, HT, ToN, JK, KE, TS, AS, YY, and YK wrote the manuscript. All authors read and approved the final manuscript.

## Conflict of Interest Statement

The authors declare that the research was conducted in the absence of any commercial or financial relationships that could be construed as a potential conflict of interest.
